# Perceptual and Neural Olfactory Similarity in Honeybees

**DOI:** 10.1371/journal.pbio.0030060

**Published:** 2005-02-22

**Authors:** Fernando Guerrieri, Marco Schubert, Jean-Christophe Sandoz, Martin Giurfa

**Affiliations:** **1**Centre de Recherches sur la Cognition Animale, CNRSUniversité Paul-Sabatier (UMR 5169), ToulouseFrance; University of London, Queen Mary CollegeUnited Kingdom

## Abstract

The question of whether or not neural activity patterns recorded in the olfactory centres of the brain correspond to olfactory perceptual measures remains unanswered. To address this question, we studied olfaction in honeybees Apis mellifera using the olfactory conditioning of the proboscis extension response. We conditioned bees to odours and tested generalisation responses to different odours. Sixteen odours were used, which varied both in their functional group (primary and secondary alcohols, aldehydes and ketones) and in their carbon-chain length (from six to nine carbons).The results obtained by presentation of a total of 16 × 16 odour pairs show that (i) all odorants presented could be learned, although acquisition was lower for short-chain ketones; (ii) generalisation varied depending both on the functional group and the carbon-chain length of odours trained; higher generalisation was found between long-chain than between short-chain molecules and between groups such as primary and secondary alcohols; (iii) for some odour pairs, cross-generalisation between odorants was asymmetric; (iv) a putative olfactory space could be defined for the honeybee with functional group and carbon-chain length as inner dimensions; (v) perceptual distances in such a space correlate well with physiological distances determined from optophysiological recordings of antennal lobe activity. We conclude that functional group and carbon-chain length are inner dimensions of the honeybee olfactory space and that neural activity in the antennal lobe reflects the perceptual quality of odours.

## Abstract 2

Training thousands of bees uncovers the chemical characteristics they use to discriminate between odours and reveals how the perception of odour correlates with specific neural activity in their brain

## Introduction

Stimulus discrimination and generalisation constitute two major abilities exhibited by most living animals. Discrimination allows treating different signals as distinct, while generalisation allows treating different but similar stimuli as equivalents [1,2,3]. Similarity along one or several perceptual dimensions determines the degree of generalisation between stimuli [2]. Determining such dimensions is fundamental for defining an animal's perceptual space. This objective remains, however, elusive in the case of the olfactory modality in which the dimensions along which odours are evaluated are not well known. Characteristics such as the functional chemical group or the carbon-chain length of a chemical substance may influence olfactory perception. It is known that at least some features of odorant molecules influence olfactory perception. For instance, some enantiomers can be discriminated by humans and nonhuman primates [4]. If and how chemical group and carbon-chain length are integrated as inner dimensions into an olfactory perceptual space remains unknown.

Vertebrate and invertebrate nervous systems show important functional as well as anatomical similarities in the way in which olfactory signals are detected and processed in their brains, particularly at the level of their first olfactory centres, the olfactory bulb in the case of vertebrates and the antennal lobe (AL) in the case of insects [5,6,7]. Insects are useful models for studying olfaction, as their behaviour heavily relies on the use of olfactory cues. The honeybee Apis mellifera is one such model in which behavioural and neurobiological studies have been performed to unravel the basis of olfaction [8,9,10,11]. Honeybee foragers are ‘flower constant' and learn and memorise a given floral species that they exploit at a time as long as it is profitable. Floral cues, among which odours play a prominent role, are then associated with nectar or pollen reward [12,13]. However, under natural conditions, the blends of volatiles emitted by floral sources vary widely in quantity and quality both in time and in space [14,15]. To cope with such changes in an efficient way, a ‘flower constant' forager should be able to generalise its choice to the same kind of floral sources despite fluctuations in their volatile emissions.

In a pioneering investigation, von Frisch [16] trained freely flying bees to visit an artificial feeder presenting several essential oils (odour mixtures). Using a set of 32 odour mixtures, von Frisch observed that after learning that a blend was associated with sucrose solution, bees tended to prefer this odour blend, but they sometimes visited other blends that were similar (to the human nose) to the rewarded one. Olfactory generalisation in honeybees was mainly studied on restrained honeybees using the conditioning of the proboscis extension reflex (PER) [17,18]. In this paradigm, harnessed honeybees are conditioned to odours associated with a sucrose reward. When the antennae of a hungry bee are touched with sucrose solution, the animal reflexively extends its proboscis to reach out towards and to lick the sucrose. Odours presented to the antennae do not usually release such a reflex in naive animals. If an odour is presented immediately before sucrose solution (forward pairing), an association is formed and the odour will subsequently trigger the PER in a subsequent unrewarded test. This effect is clearly associative and involves classical conditioning [18]. Thus, the odour can be viewed as the conditioned stimulus (CS), and sucrose solution as an appetitive unconditioned stimulus (US). Bees conditioned to individual odours or to olfactory mixtures can generalise PER to a wide range of different olfactory stimuli. Using the PER paradigm, Vareschi [19] showed that bees generalise most often between odours with similar carbon-chain lengths and between odours belonging to the same functional group. However, Vareschi conditioned odours in a differential way, with two rewarded and many unrewarded odours, so that several generalisation gradients (excitatory and inhibitory) may have interacted in an unknown way to determine the generalisation responses exhibited by the bees [19]. Using a similar approach and a restricted (6 × 6) set of odour combinations, Smith and Menzel [20] confirmed that bees generalise among odours with the same functional group, but their analysis did not detail the results obtained with individual odour combinations, thus rendering impossible the analysis of generalisation between odours with similar carbon-chain lengths. Free-flying bees trained in a differential way to a rewarded odour presented simultaneously with multiple unrewarded odours also generalise between odours with similar functional groups [21]. As for Vareschi's study [19], such an experimental design makes it difficult to interpret the generalisation responses due to unknown interactions between excitatory and inhibitory generalisation gradients.

Recently, optical imaging studies facilitated our understanding of how olfactory stimuli are detected and processed in the bee brain [22,23,24,25,26]. The first relay of the bee's olfactory system involves the ALs, which receive sensory input from the olfactory receptor neurons of the antennae within a number of 160 functional units, the glomeruli [27,28,29]. Within each glomerulus, synaptic contacts are formed with local interneurons and projection neurons (PNs). PNs send processed information from the ALs to higher brain centres such as the mushroom bodies and the lateral protocerebrum [30]. Stimulation with an odour leads to a specific spatiotemporal pattern of activated glomeruli, as shown, using in vivo calcium imaging techniques that employ fluorescent dyes to measure intracellular calcium in active neurons [22,24,31]. The odour-evoked activity patterns are conserved between individuals and constitute therefore a code [23,24]. Odours with similar chemical structures tend to present similar glomerular activity patterns [23]. Furthermore, it is believed that the neural code of odour-evoked glomerular patterns measured in the bee brain actually represent the perceptual code, although this idea was never tested directly.

In the present work, we studied behavioural olfactory generalisation, using the PER conditioning paradigm, with 16 odorants varying in two chemical features, functional group and chain length. The odours belonged to four chemical categories: alcohols with the functional group on the first or second carbon of the carbon chain (henceforth primary and secondary alcohols, respectively), aldehydes, and ketones. They possessed therefore three functional groups (alcohol, aldehyde, ketone). Their chain length ranged from six to nine carbon atoms (C6, C7, C8, and C9). The pairwise combination of 16 odours defined a 16 × 16 matrix. These odours are well discriminated by free-flying bees [21] and give consistent odour-evoked signals in optical imaging studies [23]. Using a behavioural approach, we measured similarity between odours and calculated their perceptual distances in a putative olfactory space. These perceptual distances were correlated with physiological distances measured in optical imaging experiments [23]. The correlation between both datasets was highly significant, thus indicating that odours that are encoded as physiologically similar are also perceived as similar by honeybees. Although other studies have addressed the issue of perceptual correlates of neural representations [32,33], we show for the first time that neural olfactory activity corresponds to olfactory perception defined on the basis of specific dimensions in a putative olfactory space, a finding that is of central importance in the study of the neurobiology of perception.

## Results

We trained 2,048 honeybees along three trials in which one of the 16 odours used in our experiments was paired with a reward of sucrose solution (conditioned odour). Afterwards, each bee was tested with four odours that could include or not include the trained odour.

### Acquisition Phase

The level of PER in the first conditioning trial was very low (between 0% and 8.60%) for all odours (Figure 1). All the 16 odours were learnt but not with the same efficiency. An overall (trial × odour) analysis of variance (ANOVA) showed a significant increase in responses along trials (*F*_2, 4064_ = 2215.50, *p* < 0.001) and a significant heterogeneity among odours (*F*_15, 2032_ = 8.80, *p* < 0.001). Responses to the CS in the last conditioning trial reached a level of approximately 70% for primary and secondary alcohols, 80% for aldehydes, and 61% for ketones.

**Figure 1:**
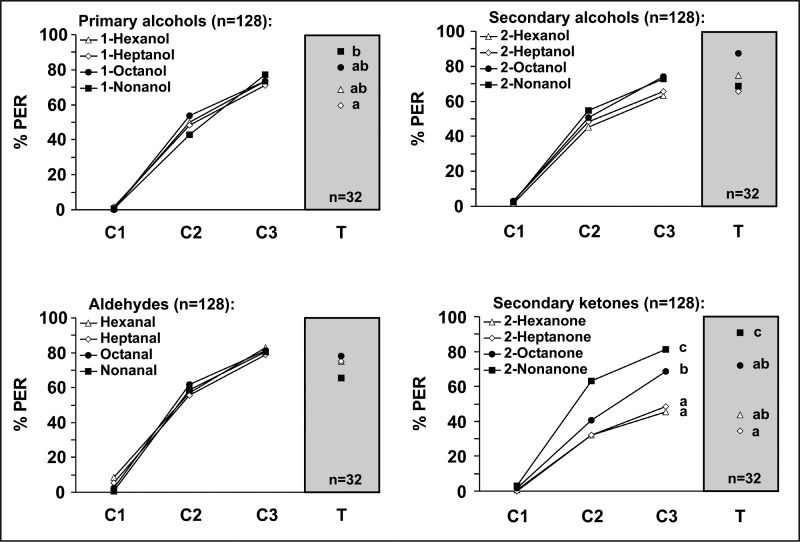
Acquisition Curves for Primary Alcohols, Secondary Alcohols, Aldehydes, and Ketones The ordinate represents the percentage of proboscis extensions to the training odour (CS). The abscissa indicates the conditioning trials (C1, C2, C3) and the test with the CS (T). The curves correspond to molecules with 6 (white triangles), 7 (white diamonds), 8 (black circles) and 9 carbons (black squares); (*n* = 128 bees for each curve). As not all 128 bees were tested with the odour used as CS, the sample size in the tests was smaller (*n* = 32). Different letters (a, b, c) indicate significant differences either between acquisition curves for different chain-length molecules (in the case of the ketones) or between test responses (post hoc Scheffé tests).

In the case of aldehydes and primary and secondary alcohols, no significant chain-length effect within functional groups was found over the whole conditioning procedure (chain length × trial ANOVA; chain-length effect for primary alcohols: *F*_3, 508_ = 0.18, *p* > 0.05; secondary alcohols: *F*_3, 508_ = 1.47, *p* > 0.05; and aldehydes: *F*_3, 508_ = 1.26, *p* > 0.05). In contrast, bees conditioned to ketones showed a significant chain-length effect in the acquisition (chain length × trial ANOVA; chain-length effect: *F*_3, 508_ = 20.00, *p* < 0.005). Scheffé post hoc comparisons showed that acquisition was significantly better for nonanone (81.25% responses in the last conditioning trial) than for all other ketones. Octanone (68.75% responses in the last conditioning trial) was also better learned than hexanone and heptanone (45.31% and 48.44% responses in the last conditioning trial, respectively) (Figure 1, bottom right). The effect over trials was significant in all cases (*p* < 0.05) as bees learned all odours.

The analysis of acquisition for each chain length separately revealed that it varied significantly depending on the functional group (functional group × trial ANOVA; C6: *F*_3, 508_ = 18.89; *p* < 0.005; C7: *F*_3, 508_ =10.78; *p* < 0.005; C8: *F*_3, 508_ = 3.84; *p* < 0.01; C9: *F*_3, 508_ = 2.73, *p* < 0.05). Scheffé post hoc comparisons generally showed that this effect was mainly due to ketones being less well learned than aldehydes and alcohols. Generally, the longer the carbon chain, the lower the heterogeneity in acquisition between functional groups. Thus, apart from short-chain ketones, all odours were learned similarly (reaching a level of acquisition between 60% and 80% in the last conditioning trial).

### Test Phase

When the conditioned odour was presented in a test (Figure 1, grey panels), the level of PER recorded corresponded mainly to that found in the last acquisition trial (McNemar tests [2 × 2 Table]: in all cases *p* > 0.05). To compare generalisation after conditioning, and because acquisition levels were heterogeneous between odours, we built a generalisation matrix in which only bees responding to the CS at the end of training (3rd conditioning trial) were considered (Figure 2). The number of individuals included in the statistical analysis varied within each ‘training odour/test odour' pair. The number of bees completing the tests varied between 17 and 28 for primary alcohols, between 13 and 29 for secondary alcohols, between 23 and 30 for aldehydes, and between 11 and 31 for ketones. The responses to the CS in the tests ranged between 70% and 100% in the generalisation matrix. All further analyses were carried out on this matrix. In the following sections, we will use the matrix data to analyse generalisation within and between functional groups, within and between chain lengths, and the asymmetries in olfactory generalisation.

**Figure 2:**
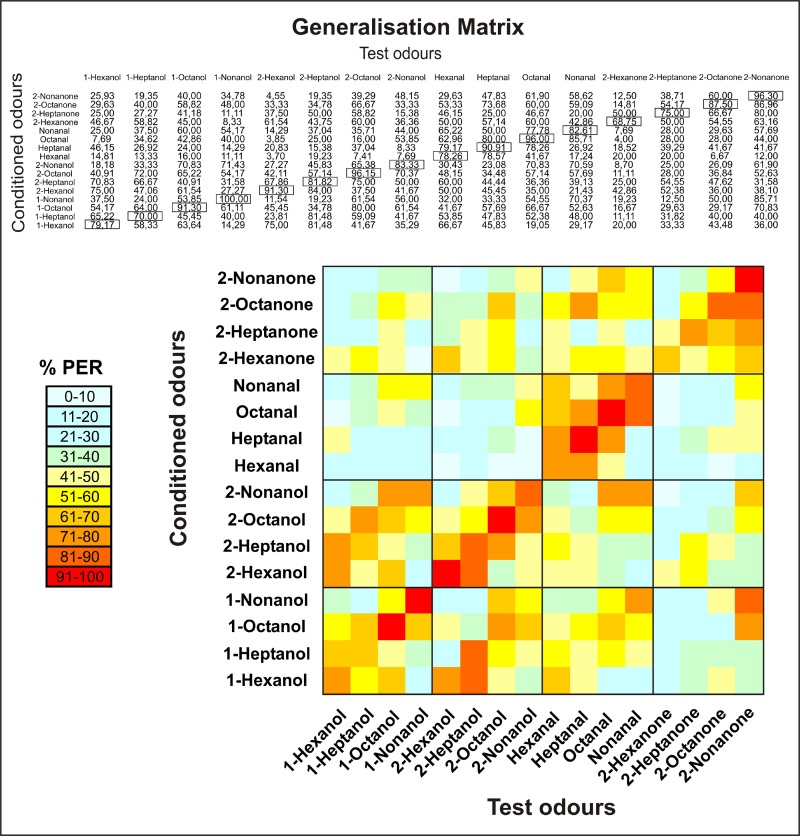
Olfactory Generalisation Matrix The generalisation matrix represents the percentage of PER in the tests performed by bees that actually learned the CS, that is, bees that responded to the CS at the third conditioning trial (*n* = 1,457). Upper part: percentages recorded. Lower part: colour-coded graphic display grouping the level of responses in ten 10% response categories. Red, maximal response; light blue, minimal response.

### Generalisation within Functional Groups

Figure 3A shows the percentage of PER to odours having different (white quadrants) or the same (grey quadrants) functional group as the conditioned odour. High levels of PER to odours different from the trained one correspond to high generalisation. In order to better visualise generalisation as depending on functional groups, we pooled all the observed responses within each quadrant of Figure 3A (i.e., not considering chain length) and calculated the resulting percentage of PER (Figure 3B). Grey bars correspond to generalisation to the same functional group; white bars correspond to generalisation to different functional groups. Generalisation mainly occurred within a given functional group (grey bars). This pattern was clearest for aldehydes (Figure 3B, 3rd row) because bees conditioned to aldehydes responded with a high probability to other aldehydes but showed lower responses to any other odour (see also the clear aldehyde “response block” in Figure 2).

**Figure 3:**
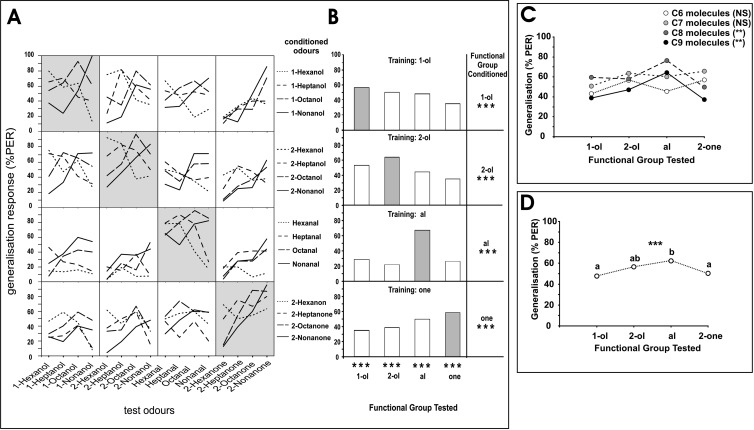
Generalisation Depending on Functional Groups (A) Data of the generalisation matrix (see Figure 2) represented as two-dimensional graphs for each conditioned odour. The right ordinate represents the CSs categorised in four functional groups, primary alcohols, secondary alcohols, aldehydes, and ketones (from top to bottom). The abscissa represents the test odours aligned in the same order as the conditioned odours (from left to right). The left ordinate represents the percentage of proboscis extensions to the test odours after being trained to a given odour. Each quadrant in the figure represents generalisation responses to one functional group after training for the same (grey quadrants) or to a different functional group (white quadrants). (B) Same data as in (A), but the observed responses within each quadrant were pooled and the resulting percentage of responses per quadrant was calculated. The abscissa and the right ordinate represent the four functional groups. The left ordinate represents the percentage of proboscis extensions to each of these groups after being trained to a given group. Grey bars correspond to grey quadrants in (A) and represent generalisation to the same functional group as the conditioned one. White bars correspond to white quadrants in (A) and represent generalisation to a functional group different from the conditioned one: 1-ol, 2-ol, al, and one mean primary alcohol, secondary alcohol, aldehyde, and ketone, respectively. Asterisks indicate significant differences along a row or a column (*p* < 0.001) (C) Within-functional group generalisation, depending on chain length. The abscissa represents the functional groups tested. The ordinate represents the percentage of proboscis extensions to the functional groups tested after being trained to a given chain-length (lines). Thus, for instance, the first point to the left for C9 molecules (black circles) represents generalisation to 1-hexanol, 1-heptanol, and 1-octanol after conditioning to 1-nonanol. A significant heterogeneity was found in within-functional group generalisation for C8 and C9 but not for C6 and C7 molecules. (D) Generalisation within-functional groups. The figure shows results from pooling the data of (C) corresponding to each functional group. Each point shows the percentage of proboscis extensions to odours of the same functional group as the conditioned odour. Within-group generalisation was significantly heterogeneous (asterisks, *p* < 0.001). Pairwise comparisons showed that generalisation within aldehydes was significantly higher than within primary alcohols or ketones and marginally higher than within secondary alcohols (different letters indicate significant differences).

We analysed within-functional group generalisation as depending on chain length (see Figure 3C). To this end we represented generalisation from C6, C7, C8, and C9 molecules having a given functional group to the other compounds having the same functional group (e.g., Figure 3C, black circle curve, first data point: generalisation to 1-hexanol, 1-heptanol, and 1-octanol after conditioning to 1-nonanol). A significant heterogeneity appeared for C8 and C9 molecules (χ^2^ = 12.60 and 14.30, respectively, *p* < 0.01 in both cases, *n* = 67–85) but not for C6 and C7 molecules (*p* > 0.05). In the case of C8 and C9 molecules, generalisation was significantly higher within aldehydes (*p* < 0.05).

When comparing within-group generalisation over all four functional groups (Figure 3D), a significant heterogeneity appeared (χ^2^ = 14.40, *df* = 3, *p* < 0.01, *n* = 276–316). Pairwise comparisons (using a corrected threshold for multiple comparisons: α′ = 0.017) showed that generalisation within aldehydes was significantly higher than within primary alcohols (χ^2^ = 11.80, *df* = 1, *p* < 0.0006) and ketones (χ^2^ = 9.90, *df* = 1, *p* < 0.005) and close to significance in favour of aldehydes when compared to secondary alcohols (χ^2^ = 4.40, *df* = 1, 0.017 < *p* < 0.05).

### Generalisation within Chain Lengths

Figure 4A shows the generalisation responses of bees to odours having different (white quadrants) or the same (grey quadrants) chain length as the conditioned odour. In order to better visualise generalisation as depending on chain length, we pooled all the observed responses within each quadrant of Figure 4A and calculated the resulting percentage of PER (Figure 4B). Grey bars correspond to generalisation to the same chain length; white bars correspond to generalisation to different chain lengths. Generalisation was highest in the case of odours with the same or similar chain length.

**Figure 4:**
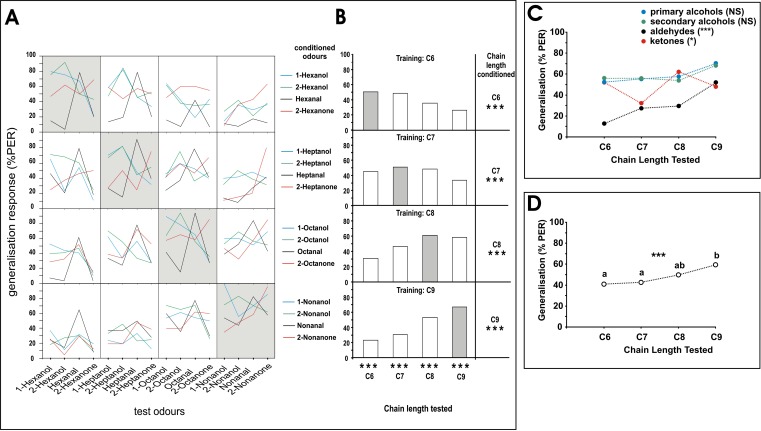
Generalisation Depending on Chain Length (A) Data of the generalisation matrix (see Figure 2) represented as two-dimensional graphs for each conditioned odour. The right ordinate represents the CSs categorised in four chain lengths, C6, C7, C8, and C9 molecules (from top to bottom). The abscissa represents the test odours aligned in the same order as the conditioned odours (from left to right). The left ordinate represents the percentage of proboscis extensions to the test odours after being trained for a given odour. Each quadrant in the figure represents generalisation responses to one chain length after training for the same (grey quadrants) or to a different chain length (white quadrants). (B) Same data as in (A), but the observed responses within each quadrant were pooled and the resulting percentage of responses per quadrant was calculated. The abscissa and the right ordinate represent the four chain-length categories. The left ordinate represents the percentage of proboscis extensions to each of these categories after being trained for a given chain-length category. Grey bars correspond to grey quadrants in (A) and represent generalisation to the same chain length as the conditioned one. White bars correspond to white quadrants in (A) and represent generalisation to a chain length different from the conditioned one: C6, C7, C8, and C9 mean chain length of 6, 7, 8, and 9 carbons, respectively. Asterisks indicate significant differences along a row or a column (*p* < 0.001). (C) Within chain-length generalisation as depending on functional group. The abscissa represents the chain lengths tested. The ordinate represents the percentage of proboscis extensions to the same chain length after being trained to a given functional group (lines). Thus, the first point to the left for ketones (red circles) represents generalisation to 1-hexanol, 2-hexanol, and hexanal after conditioning to 2-hexanone; the second point represents generalisation to 1-heptanol, 2-heptanol, and heptanal after conditioning to 2-heptanone. A significant heterogeneity was found in within-chain-length generalisation for aldehydes and ketones. (D) Generalisation within-chain lengths. The figure results from pooling the data of (C) corresponding to each chain length. Each point shows the percentage of proboscis extensions to odours of the same chain length as the conditioned odour. Within-chain-length generalisation was significantly heterogeneous (asterisks, *p* < 0.001). Pairwise comparisons showed that generalisation within C9 molecules was significantly higher than within C7 and C6 molecules and marginally higher than within C8 molecules (different letters indicate significant differences).

We analysed within-chain length generalisation as depending on functional group (Figure 4C). To this end we represented generalisation from primary alcohols, secondary alcohols, aldehydes, or ketones of a given chain length to the other compounds having the same chain length (e.g., Figure 4C, red circle curve, first data point: generalisation to 1-hexanol, 2-hexanol, and hexanal after conditioning to 2-hexanone). Generalisation within-chain length was generally higher for longer than for shorter chain lengths. This effect was significant for aldehydes (χ^2^ = 28.70, *df* = 3, *p* < 0.01, *n* = 75–80) but not for primary and secondary alcohols (χ^2^ = 5.20 and 3.4, *df* = 3, *p* > 0.05, *n* = 67–73 and *n* = 61–66, respectively). For ketones, a significant heterogeneity was found (χ^2^ = 10.00, *df* = 3, *p* < 0.05, *n* = 40–79), but generalisation was more important between C8 than between C7 molecules. The generalisation corresponding to other chain lengths fell in between.

When comparing within-chain length generalisation over all four chain-length groups (Figure 4D, i.e., not considering functional group), a significant heterogeneity appeared χ^2^ = 23.2, *df* = 3, *p* < 0.001, *n* = 247–293). Pairwise comparisons (using a corrected threshold for multiple comparisons: α′ = 0.017) showed that within-chain length generalisation was significantly higher within C9 than within C6 (χ^2^ = 18.50, *df* = 1, *p* < 0.0001) and C7 molecules (χ^2^ = 15.00, *df* = 1, *p* < 0.0001). Generalisation within C8 molecules was close to significance when compared to generalisation within C9 molecules (χ^2^ = 5.00, *df* = 1, 0.017 < *p* < 0.05), and it was significantly higher than generalisation within C6 molecules (χ^2^ = 4.3, *df* = 1, 0.017 < *p* < 0.05).

### Generalisation between Functional Groups

To analyse generalisation between groups, we took into account the responses to functional groups different from the conditioned one (see white bars in Figure 3B). Bees showed heterogeneous patterns of generalisation (all vertical and horizontal comparisons in Figure 3B were significant: χ^2^ > 37.70, *df* = 3, *p* < 0.001, in all eight cases). We found high between-group generalisation for primary and secondary alcohols: bees conditioned to secondary alcohols responded preferentially to primary alcohols, somewhat less to aldehydes, and even less to ketones (see Figures 3A and 3B, second row). A similar but less obvious response gradation was found for bees conditioned to primary alcohols Figures 3A and 3B, first row). In fact, the overall generalisation patterns were very similar for primary and secondary alcohols sharing the same chain length (see, for instance, the very close relationship between the two sets of blue [primary alcohol] and green curves [secondary alcohols] in Figure 4A).

As indicated before, bees conditioned to aldehydes generalised very little to odours belonging to other functional groups (see Figure 3B, third row). Contrarily, bees conditioned to other functional groups highly generalised to aldehydes (see third column ‘al' in Figure 3B). This shows that generalisation between aldehydes and odours belonging to other functional groups was asymmetrical. The topic of asymmetric generalisation will be considered below in more detail.

### Generalisation between Chain Lengths

To analyse generalisation between chain lengths, we took into account the responses to chain lengths that differed from the conditioned one (see white bars in Figure 4B). In general, responses to molecules with different chain lengths followed a clear decreasing gradient, depending on the difference in the number of carbon atoms between the molecules considered (see Figure 4B; all horizontal and vertical comparisons were significant, χ^2^ > 16.3, *df* = 3, *p* < 0.001 in all eight cases). For instance, when conditioned to a C9 molecule (see Figure 4B, fourth row), bees responded in 53%, 31%, and 23% of the cases to C8, C7, and C6 molecules, respectively, while they responded to C9 molecules in 67% of the cases. This gradient was also evident when generalisation took place between functional groups: for instance, after training with 2-nonanol (see Figure 3A, second row), the response of bees to odours of different functional groups (solid lines in white boxes) always followed a similar decreasing tendency with the same (C9) or similar (C8) chain length on top.

### Asymmetry in Olfactory Generalisation

As previously mentioned, some groups like aldehydes induced asymmetrical cross-generalisation (i.e., bees responded less to other functional groups after training for aldehydes than to aldehydes after training for other functional groups). We analysed this asymmetrical generalisation and built an asymmetry matrix (Figure 5A). To this end, we calculated for each odour pair (A and B) the difference (in percentage) between generalisation from A to B and generalisation from B to A. Such differences were ranked in 10% categories from −55% to 55%. White boxes indicate no asymmetries. Blue shades in Figure 5A indicate that cross-generalisation was biased towards odour A (i.e., conditioning to A resulted in lower generalisation to B while conditioning to B resulted in higher generalisation to A); red shades indicate that cross-generalisation was biased towards odour B (i.e., conditioning to A resulted in higher generalisation to B while conditioning to B resulted in lower generalisation to A). This representation showed that some odours induced generalisation while other odours diminished it. For instance, hexanal was well learnt but induced low generalisation to other odours, except to other aldehydes. On the other hand, bees conditioned to other odours very often generalised to hexanal. Thus, a clear blue row (or a red column) corresponds to hexanal in the asymmetry matrix. Conversely, 2-hexanone induced high generalisation to other odours but received few responses as a test odour. Thus a red row (or a blue column) corresponds to 2-hexanone in the asymmetry matrix. Most odours, however, showed little or no asymmetry. Figure 5B presents the mean asymmetry found for each training odour. In six cases, the mean asymmetry deviated significantly from zero, which represents a theoretically perfect symmetry (*t*-test). Two odours (red bars) significantly induced generalisation (2-hexanone and 2-hexanol, *t*-test, *df* = 14, *p* < 0.001 and *p* < 0.01, respectively), while four odours (blue bars) diminished it significantly (hexanal, heptanal, and octanal, and 2-nonanone, *t*-test, *df* = 14, *p* < 0.001 for the former and *p* < 0.01 for the three latter odours).

**Figure 5:**
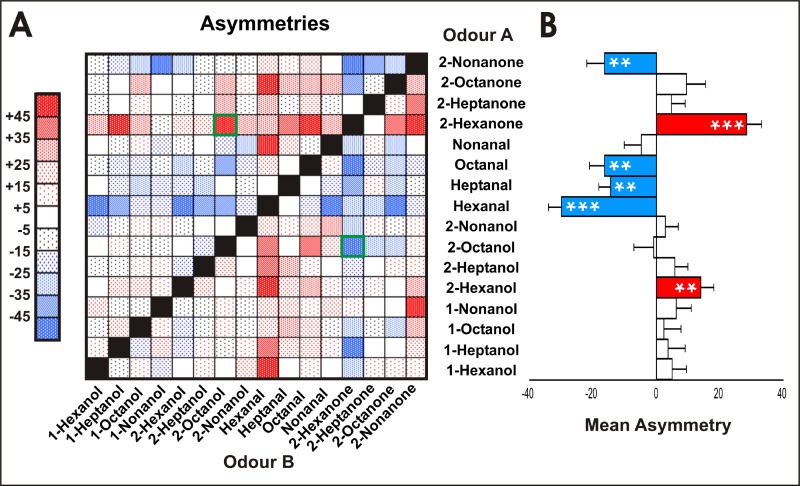
Asymmetric Generalisation between Odours (A) The asymmetry matrix depicts asymmetric cross-generalisation between odours. For each odour pair (A and B), the difference (percentage) between generalisation from A to B and generalisation from B to A was calculated. Such differences were ranked in 10% categories varying from blue (−55%) to red (55%). Blue shades indicate that cross-generalisation was biased towards odour A (i.e., conditioning to A resulted in lower generalisation to B, while conditioning to B resulted in higher generalisation to A); red shades indicate that cross-generalisation was biased towards odour B (i.e., conditioning to A resulted in higher generalisation to B, while conditioning to B resulted in lower generalisation to A). For this reason, each odour pair (A and B) appears twice in the matrix, once in the upper-left of the black diagonal line, and once in the lower-right of the black diagonal line, with opposite values. See, for example, the two cells outlined in green for the pair 2-hexanone/2-octanol. (B) Mean generalisation induced or diminished by each odour A in (A). Each bar represents the mean asymmetry of the respective horizontal line in the asymmetry matrix. Red bars show that an odour induced more generalisation than it received, while blue bars show the opposite. Significant generalisation asymmetries were found in six out of 16 cases (**, *p* < 0.01; ***, *p* < 0.001).

### Olfactory Space

In order to define a putative olfactory space for the honeybee, we performed a principal component analysis (PCA) on our data to represent in a limited number of dimensions the relative relationships between odorants in a 16-dimension perceptual space (Figure 6A). The first three factors represented 31%, 29%, and 15% of overall variance in the data (total of the first three factors: 75%). The analysis showed a clear organisation of odours depending on their chemical characteristics. First, chain length was very clearly represented by the first factor (see upper-right graph in Figure 6A), from C6 to C9 molecules from the right to the left. On the other hand, the chemical group was mostly represented by factors 2 and 3. Whereas factor 2 separated mostly aldehydes from alcohols, with ketones falling between them, factor 3 segregated ketones from all other odours (lower-right graph, Figure 6A). None of these factors separated primary and secondary alcohols. This analysis indicates that the chemical features of molecules (chain length and functional group), which are sometimes thought of as artificial perceptual (psychophysical) dimensions determined by experimenters [34] can be considered as true inner dimensions of the bees' perceptual space. Cluster analyses performed on the data segregated odours mostly according to their chain length. In the first group (Figure 6B, upper part), we found two subgroups, short-chain alcohols (C6 and C7, primary and secondary alcohols) and short-chain ketones (C6 to C8). On the other hand (Figure 6B, lower part), three clear subgroups were formed: short-chain aldehydes (C6 and C7), long-chain alcohols (C8 and C9, primary and secondary alcohols), and a last group with long-chain aldehydes (C8 and C9) and 2-nonanone. Very similar results were obtained using Euclidian or city-block metrics.

**Figure 6:**
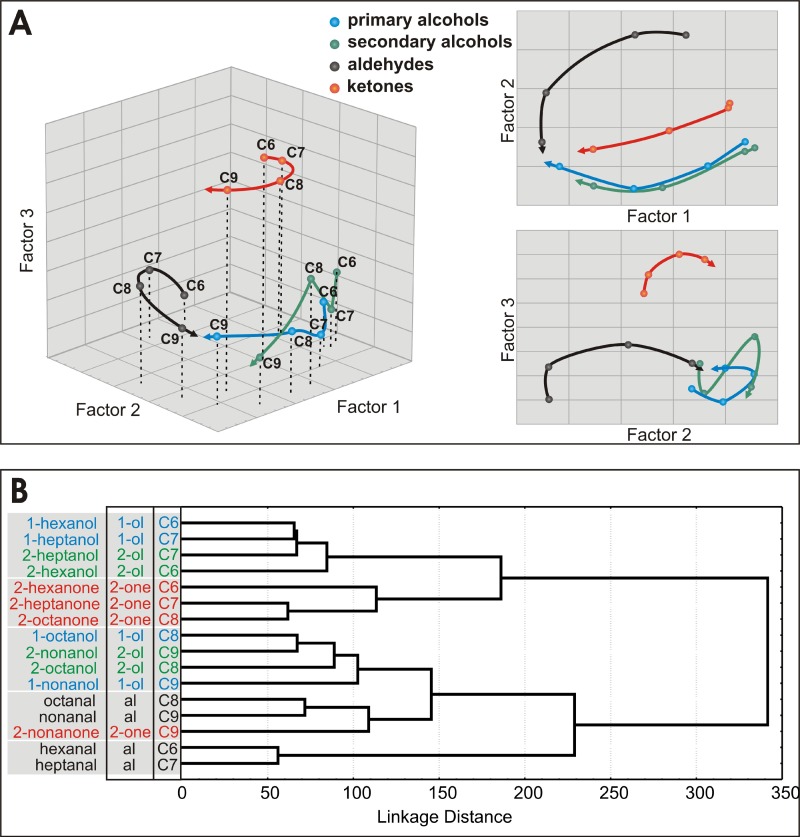
A Putative Honeybee Olfactory Space (A) Left: The olfactory space is defined on the basis of the three principal factors that accounted for 76% of overall data variance after a PCA performed to represent the relative relationships between odorants. Primary alcohols are indicated in blue, secondary alcohols in green, aldehydes in black, and ketones in red. Different chain-lengths are indicated as C6, C7, C8, and C9, which corresponds to their number of carbon atoms. For each functional group, arrows follow the increasing order of carbon-chain lengths. Right: Chain length was very clearly represented by factor 1. C6 to C9 molecules are ordered from right to left. The chemical group was mostly represented by factors 2 and 3. Whereas factor 2 separated mostly aldehydes from alcohols, with ketones falling between them, factor 3 separated ketones from all other odours. None of these three factors separated primary and secondary alcohols. (B) Euclidean cluster analysis. The analysis separated odours mostly according to their chain length. Linkage distance is correlated to odour distances in the whole 16-dimension space. The farther to the right two odours/odour groups are connected, the higher the perceptual distance between them (odour colour codes are the same as in [A]).

### Correlation between Optophysiological and Behavioural Measures of Odour Similarity

We asked whether optophysiological measures of odour similarity, obtained using calcium imaging techniques at the level of the honeybee AL [22,23,24,35], correspond to perceptual odour similarity measures as defined in our putative honeybee olfactory space. We thus calculated the Euclidian distance between odour representations in our 16-dimension “behavioural” space for all odour pairs (120 pairs). We then calculated distances between odours in optical imaging experiments, using the odour maps by Sachse et al. [23]. A correlation analysis was performed between both datasets. This analysis was possible because both the study by Sachse et al.[23] and our study used the same set of odours delivered under the same conditions. Figure 7A presents the correlation obtained, including all 120 odour pairs. Both sets of data were highly significantly correlated (*r* = 0.54, *t*_118_ = 7.43, *p* < 2.10^–10^), a result that shows that odours, which were found to be physiologically similar in the optical imaging study, were also evaluated as similar in behavioural terms. Note, however, that data points cluster quite broadly around the main trend line, showing that many exceptions were found. In order to use a more exact measure of physiological odour similarity, we used the correlation results between primary and secondary alcohol maps provided by Sachse et al. [23]. By correlating this more exact value of physiological similarity with our behavioural data, we also found a highly significant relationship between physiological and behavioural data (Figure 7B; *r* = 0.82, *t*_26_ = 7.83, *p* < 7.10^–8^). The correlation coefficient achieved with this second method was significantly higher than that achieved with the first method (*Z* = 2.52, *p* < 0.05). A better fit between the two datasets was thus found, although outliers were still present in the data. These two analyses show that optophysiological and behavioural measures of odour similarity correlate well using the methods described here. Thus, in the case of the honeybee, olfactory neural activity corresponds to olfactory perception.

**Figure 7:**
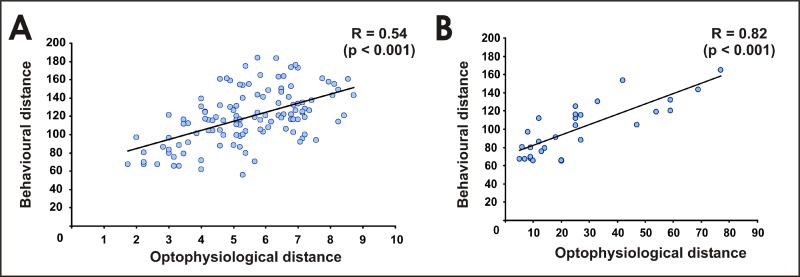
Correspondence between Perceptual and Physiological Odour Similarity (A) Correlation between optophysiological measures of odour similarity (carried out using calcium imaging recordings [23]) and our behavioural measures of odour similarity. Euclidian distance between odour representations in our 16-dimension “behavioural” space for all odour pairs (120 pairs, x axes) and distances between odours in optical imaging experiments, using the odour category maps displayed by Sachse et al. [23] (also 120 pairs, y axes) were calculated. This correlation, including all 120 odour pairs, was highly significant (*r* = 0.54, *p* < 0.001). Odours found to be similar in the optical imaging study were also similar in the behaviour. Data points cluster quite broadly around the main trend line, showing that many exceptions were found. (B) Correlation between measures of optophysiological similarity carried out using the optical imaging technique [23] and our behavioural measure of odour similarity. Using the exact data given for primary and secondary alcohols [23], a much better correlation between the two datasets was achieved than in (A) (*r* = 0.82, *p* < 0.001), although outliers were still found in the data.

## Discussion

In the present work, we have studied perceptual similarity among odorants in the honeybee, using an appetitive-conditioning paradigm, the olfactory conditioning of the PER [17,18]. We showed that all odorants presented could be learned, although acquisition was lower for short-chain ketones. Generalisation varied, depending both on the functional group and on the carbon-chain length of odours trained. Generalisation was very high among primary and secondary alcohols, being high from ketones to alcohols and aldehydes and low from aldehydes to all other tested odours; thus, in some cases, cross-generalisation between odorants was asymmetric. Some odours, like short-chain ketones or aldehydes, induced more asymmetries than other odours. Higher generalisation was found between long-chain than between short-chain molecules. Functional group and carbon-chain length constitute orthogonal inner dimensions of a putative olfactory space of honeybees. Perceptual distances in such a space correlate well with physiological distances determined from optophysiological recordings performed at the level of the primary olfactory centre, the AL [23] such that olfactory neural activity corresponds to olfactory perception.

Previous studies have attempted to describe olfactory generalisation in honeybees and to study structure–activity relationships [19,20,36,37,38]. These studies generally supported the view that generalisation mainly happens when odours belong to the same chemical group. Moreover, they also suggested that the rules underlying olfactory learning and perception of different chemical classes [20] or of particular odorants (e.g., citral [20,37]) may vary. However, these studies used differential training, thus inducing several generalisation gradients (excitatory and inhibitory) that make the interpretation of generalisation responses difficult [21,36]. Furthermore, these studies were carried out on a rather discrete number of odour pairs [37], did not detail the results obtained with individual odour combinations [20], or used a very reduced number of bees per conditioned odour ([21]; two bees per odorant).Thus, the present study is the first one to provide (i) generalisation data based on absolute conditioning (i.e., only one odour conditioned at a time), (ii) a systematical test of all odour combinations, (iii) robust sample sizes for each experimental situation, and (iv) important generalisation gradients. These are in our view crucial prerequisites to describe odour perception and similarity in a precise way.

### Chemical Group and Chain Length

Several studies in other species have shown the importance of functional group and carbon-chain length of the odour molecules for behavioural responses to odours. Differences in the response between molecules of diverse aliphatic and aromatic homologue odour classes (i.e., differing in functional group, chain length, and overall molecule form) were investigated in moths [39,40], cockroaches [41], rats [42], squirrel monkeys [4,43] and humans [38,44,45]. These studies show that both functional group and chain length affect the perceived quality of an odorant. Concerning chain length, the greater the difference in the number of carbons between odours, the easier the discrimination and the lower the generalisation ([21,40,42,44] and present study).

In our study, both chemical group and chain length of odour molecules determined the bees' generalisation responses. Bees mostly generalised to other odours when these shared the same functional group. This effect was observed for all functional groups (see Figure 3B) but was strongest for aldehydes. Other studies have found that aldehydes induced high within-group generalisation [20,21,36]. Thus, aldehydes may represent a behaviourally relevant chemical class for honeybees. Between-functional group generalisation depended on the functional group considered. It was high between primary and secondary alcohols, which appear therefore perceptually similar to the bees, and low between other chemical groups. Bees clearly generalised between odours that shared the same chain length. Increasing chain length promoted generalisation. Moreover, generalisation to other chain lengths decreased if the difference in the number of carbons between odours increased. This suggests a perceptual continuum between different chain lengths (but see below). Thus, the chemical structure of the odorants is critical for determining the amount of generalisation.

### A Putative Olfactory Space for the Honeybee

We found that the two controlled physical characteristics of odour molecules used in this study, functional group and chain length, correspond to internal dimensions in the bees' olfactory perceptual space such as the three most important factors extracted in our PCA analysis, one mainly represented chain length and the other two were mostly influenced by functional group. Cluster analyses allowed separating odours in clusters according to their functional groups and their chain length. Interestingly, C6 and C7 molecules and C8 and C9 molecules were mainly grouped together, so that, for instance, all short-chain primary and secondary alcohols were grouped on one side, and all long-chain alcohols on the other side. The same happened for aldehydes, and in a different way for ketones (C9 separated from the rest). This discrepancy suggests that, although chain length appears mostly as a perceptual continuum in the PCA analysis, there may be a perceptual “jump” between short-chain and long-chain molecules.

### Neural Bases of Odour Perception

Both in vertebrates and in invertebrates, studies quantifying the neural responses to structurally similar odours in the first relay of the olfactory pathway have been performed (olfactory bulb: e.g., [46,47,48,49]; AL: [23,50]). These studies show that activity patterns are more similar when the difference in the number of carbons between molecules is small. It was hypothesised that such a physiological similarity is the basis for olfactory discrimination and generalisation as measured behaviourally. This has indeed been reported for mucosal activity in mice [51], electrical mitral cell activity [42], and/or radiolabelled 2-deoxyglucose uptake in the rat olfactory bulb [32]. Also, in *Manduca sexta,* qualitative similarities were observed between the degree of behavioural generalisation according to chain length [40] and the degree of overlap between electrophysiological temporal patterns of activity across AL neurons [50].

Several correspondences, but also discrepancies, can be found between our behavioural results and the physiological results obtained at the level of the bee AL [23]. First, within the regions of the AL accessible to optical imaging (about 25% of the glomeruli), patterns of glomerular activity for different odours are highly dependent on chain length, but much less so on chemical group. Thus, most active glomeruli respond to several functional groups as long as the chain length corresponds, but respond differentially to different chain lengths. Glomeruli T1–28 and T1–52 are specialised in short-chain molecules (respectively C5–C7 and C6–C7), whilst glomeruli T1–33 and T1–17 are specialised in long-chain molecules (respectively C7–C9 and C8–C9). These glomeruli also respond to most functional groups but in a graded way. For instance, glomerulus T1–17 responds more to alcohols in the intermediate range than to aldehydes or ketones, whereas T1–52 generally responds more to ketones in the short range, more to aldehydes in the long range, and overall little to alcohols. No individual glomerulus was found that responds specifically to a chemical group. However, it should be kept in mind that some regions of the ALs are not yet accessible to calcium imaging techniques (about 75% of the lobe; see below). Thus, a possible explanation is that glomeruli responding to specific chemical groups (or with responses more dependent on chemical groups than on chain length) were not imaged.

Second, primary and secondary alcohols induce extremely similar activation patterns in the AL, but subtle differences could be found, so that for a given chain length, the representation of a secondary alcohol was between that of the primary alcohol of the same chain length and that with one less carbon atom (see Figure 6B in Sachse et al. [23]). We found a similar arrangement of alcohol representations, with primary and secondary alcohols alternating on a common axis (see Figure 6A).

Third, optical imaging data showed that higher chain lengths support more similarity between patterns (see Figure 6C in Sachse et al. [23]). Our finding that longer chain lengths induce more generalisation agrees with the imaging data. These last two points suggest that the general rules governing odour similarity at the neural and the behavioural level are similar.

### The Correspondence between Perceptual and Physiological Odour Similarity

We aimed at comparing behavioural and physiological data in a more precise way, using correlation analyses between our behavioural similarity matrix, in which distances between two odour points represent psychological distances between stimuli, and a physiological similarity matrix obtained from optophysiological recordings of glomerular activation patterns [23]. Comparing distances between odours in these two matrixes resulted in a good correlation. This means that glomerular activity patterns recorded in the brain could predict behavioural responses and vice versa.

The optophysiological dataset of Sachse et al. [23] has nevertheless some limitations with respect to the objectives of our work: (i) bath application measurements of AL activity using calcium green as a dye [23] record the combined activity of several neuronal populations of the AL, among which primary-afferent activity seems to have the most important contribution [52]; (ii) such measurements survey only the dorsal part of the AL, which constitutes 25% of the neuropile studied; and (iii) learning alters odour representations in the AL [35,53,54] such that there could be a mismatch between our data collected after olfactory conditioning and the dataset of Sachse et al. [23], which was obtained from naive bees.

With respect to the first point, it could be argued that the AL circuitry transforms the primary-afferent representations of odours [25] such that recordings where primary-afferent receptor activity is predominant are not very useful for evaluating optophysiological similarity. However the very fact that we found a significant correlation between our behavioural data and the imaging data by Sachse et al. [23], strongly suggests that the perceptual quality of odorants mostly appears at the peripheral level. Clearly, this correlation was not perfect, and odour quality is most probably refined by further processing within the AL, and/or at higher stages of the olfactory pathway, such as in the mushroom bodies or the lateral protocerebrum. In honeybees, new methods have been developed, which allow recording selectively the activity of the efferent PNs [25]. However, the two studies published using this method [25,26] do not provide an extensive odorant matrix as that provided by Sachse et al. [23]. In this sense the study on which we based our correlation analysis is certainly the only one of its kind published to date. However, in the future, a careful comparison of our behavioural data with both bath-applied imaging data emphasising receptor neuron input (as done here) and selective imaging of PNs would be extremely helpful in understanding to what extent AL processing shapes odour perceptual quality.

With respect to the second point, calcium imaging recordings of AL activity are certainly limited to the dorsal part of the AL, which is the region accessible when the head capsule is opened in order to expose the brain for recordings. This is an inherent limitation of the method that the use of two-photon microscopy during calcium imaging measurements will soon allow us to overcome, as shown already by recordings obtained in the fruit fly Drosophila melanogaster [55].

Finally, with respect to the third point, it is known that learning alters odour representations in the AL, when bees are trained in a differential conditioning procedure, with one odour rewarded and another odour unrewarded [53]. This is not the conditioning procedure used in our work, which was absolute (only one odour rewarded at a time). In the bee, changes in the olfactory code due to absolute conditioning seem to be difficult to detect (C. G. Galizia, personal communication), such that this point may not be so critical for our correlation analysis. In any case, if there are changes in odour representations due to conditioning, recording glomerular activity patterns after conditioning would only improve our correlation analyses.

### Generalisation Asymmetries between Odours

We have found a number of asymmetries in olfactory cross-generalisation, with bees responding more to odour B after learning odour A than in the reverse situation. Previous studies have observed such a phenomenon, but it was mostly related to olfactory compounds with pheromonal value (aggregation pheromone citral [20,37] and alarm pheromones 2-heptanone and isoamyl acetate [56]). In the present study, we found that six out of the 16 odours used induced significant generalisation asymmetries over the whole matrix; none of these six odours was related to any known pheromone (see Table 1). Generalisation asymmetries seem to be a general feature of honeybee olfaction.

**Table 1:**
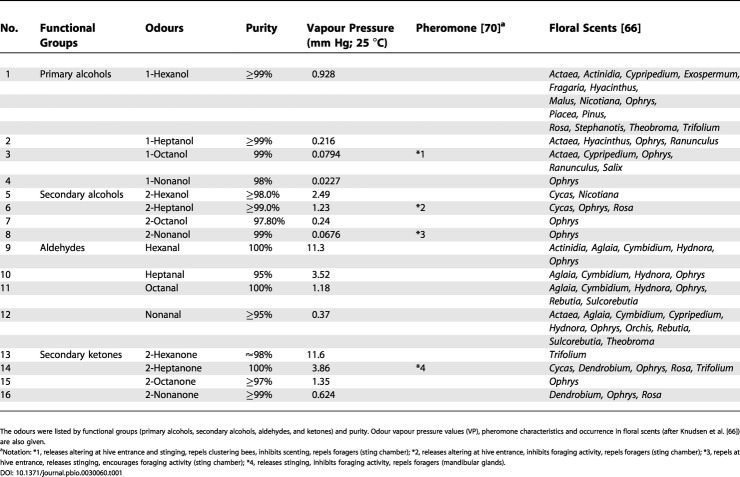
Chemical and Biological Characteristics of the Odours Used

The odours were listed by functional groups (primary alcohols, secondary alcohols, aldehydes, and ketones) and purity. Odour vapour pressure values (VP), pheromone characteristics and occurrence in floral scents (after Knudsen et al. [66]) are also given

^a^Notation: *1, releases altering at hive entrance and stinging, repels clustering bees, inhibits scenting, repels foragers (sting chamber); *2, releases altering at hive entrance, inhibits foraging activity, repels foragers (sting chamber); *3, repels at hive entrance, releases stinging, encourages foraging activity (sting chamber); *4, releases stinging, inhibits foraging activity, repels foragers (mandibular glands)

Odour concentration can affect stimulus salience. In our work, generalisation asymmetries could not be directly explained by differences in odour concentration (through differences in vapour pressure), because, for instance, the two odours with the highest vapour pressure in our sample (2-hexanone and hexanal) produced totally opposite results: 2-hexanone induced important generalisation, while hexanal strongly reduced generalisation. Also, although we used 16 different odours with a range of different vapour pressures, we found that acquisition was very similar for most odours, except for the short-chain ketones, which were less easily learned. This suggests that almost all odours used had a good salience for bees. Wright and Smith [57] studied the effect of odour concentration in generalisation in honeybees. They found that discrimination increased with concentration for structurally dissimilar odours but not for similar odours. Further experiments using odorants at different concentrations should be carried out to determine the effect of odour concentration on generalisation asymmetries.

Generalisation asymmetries could be due to innate or experience-dependent differences in the salience of odours for honeybees, such that more salient odours would induce higher generalisation than less salient odours. This interpretation implies that most aldehydes (hexanal, heptanal, and octanal) are highly salient odours for honeybees, because aldehydes showed a clear “functional group” effect, which could reveal a certain bias of the olfactory system towards these odours. Ketones, on the other hand, showed a heterogeneous effect, as 2-hexanone seemed to have a low salience (it was not well learnt) and induced a high generalisation to other odours, while 2-nonanone consistently reduced generalisation to other odours. In the group of alcohols, only 2-hexanol induced generalisation to other odours. Therefore, only aldehydes showed a clear group effect on generalisation asymmetry. This effect could be due to innate odour preferences [58,59] or to previous odour exposure within the hive [60,61]. Innate odour preferences could be related to natural, floral odours that were more consistently associated with food resources [20,62]. It is thus important to investigate whether or not such ecological trends exist in the natural flora associated with the honeybee and whether or not other bee species also present such clear biases, in particular towards aldehydes.

Conversely, asymmetries could be the result of the conditioning procedure. This would be the case if conditioning modifies odour representation in an asymmetric way. Indeed, experience-induced modifications of odour representations have been found at the level of the honeybee AL. Thus, odour-evoked calcium signals in the AL can be modified by elemental [53] and nonelemental olfactory learning paradigms [35] such that the representations of odours that have to be discriminated become more distinct and uncorrelated as a result of learning. In the fruit fly D. melanogaster, new glomeruli become active after olfactory learning [54], while in the moth M. sexta new neuronal units in the AL are recruited after olfactory learning [63]. These elements suggest that modifications of odour representation after learning two different odours could indeed be asymmetrical: if, for instance, the neuronal representation of A after conditioning becomes A′, which is slightly farther away from B than A in the bee's olfactory space, and if the perceptual representation of B becomes B′ after conditioning, which is closer to A than B, then bees would show less generalisation in behavioural tests from A to B than from B to A. On the level of the AL network, glomeruli are connected via lateral inhibitory interneurons [25,64,65]. Due to this, glomerular activation by an odour A will transiently inactivate parts of the network and possibly parts encoding a subsequent odour B. Optical imaging experiments have shown that inhibition between glomeruli may be asymmetric [25]. In our case, glomeruli activated by odour A may inhibit glomeruli coding for odour B, while glomeruli coding for odour B may not inhibit those coding for odour A. In this hypothesis, asymmetric cross-generalisation could reflect a sensory phenomenon. Nevertheless, we believe that inhibitions at the level of the AL are rather short-lived such that a purely sensory priming effect seems improbable. If, however, the strength of lateral inhibitions between glomeruli can be modified by learning as proposed by Linster and Smith [65], then asymmetrical generalisation would come from the fact that inhibitory lateral connections are modified. In order to determine the physiological mechanisms underlying asymmetrical cross-generalisation and the possible role of AL networks in it, future work will aim at visualising the evolution of glomerular activity patterns during and after olfactory conditioning with odours that showed asymmetries in our study.

### Conclusion

We have shown that the two odorant physical dimensions that varied in our study, functional group and chain-length, correspond to internal dimensions of the bees' olfactory space. Generalisation was mainly due to these two characteristics with generalisation within functional group being more important. Such generalisation was particularly high for aldehydes, a fact that suggests that these odours may have an intrinsic value for bees. Generalisation between functional groups was mostly found between primary and secondary alcohols. Furthermore, a gradient in generalisation was found with respect to chain length. Asymmetric cross-generalisation was found in the case of certain odorants. Such asymmetries were neither strictly linked to chain length nor to functional group, but depended on particular odorants.

The 16 odours used in our work represent a small part of the odorants that bees may encounter in nature (see Knudsen et al. [66]). For a complete description of the bees' olfactory perceptual space, more odours having other molecular features have to be studied. New dimensions in the bees' perceptual space could then be found.

Finally, and most important, the perceptual distance between odours can be predicted on the basis of the differences in the patterns of glomerular activation in the first relay of the olfactory pathway: the AL, and vice versa. This emphasises the relevance of studying activity patterns in the brain in imaging studies and trying to relate them to perceptual tasks. Our work shows that this objective, which is at the core of cognitive neurosciences, can be achieved using an invertebrate model such as the honeybee.

## Materials and Methods

### 

#### Insects

Every experimental day, honeybees were captured at the entrance of an outdoor hive and were cooled on ice for 5 min until they stopped moving. Then they were harnessed in small metal tubes in such a way that only the head protruded. The mouthparts and the antennae could move freely. Harnessed bees were left for 3 h in a resting room without disturbance. Fifteen minutes before starting the experiments, each subject was checked for intact PER by lightly touching one antenna with a toothpick imbibed with 50% (w/w) sucrose solution without subsequent feeding. Extension of the proboscis beyond the virtual line between the open mandibles was counted as PER. Animals that did not show the reflex were not used in the experiments.

#### Stimulation apparatus

The odours were delivered by an odour cannon, which allowed the presentation of up to seven different odours, and a clean airstream [67]. Each odour was applied to a filter paper placed within a syringe (see below) that was connected to the cannon. An airstream was produced by an air pump (Rena Air 400, Annecy, France) and directed to the relevant syringes with electronic valves (Lee Company, Voisins-le-Bretonneux, France) controlled by the experimenter via a computer. In the absence of odour stimulation, the airstream passed through a syringe containing a clean filter paper piece (clean airstream). During odour stimulation, the airstream was directed to a syringe containing a filter paper loaded with odour. After a 4-s stimulation, the airstream was redirected to the odourless syringe until the next stimulation.

#### Stimuli

Sixteen odours (Sigma Aldrich, Deisenhofen, Germany) were used in our work as CS and test stimuli (see Table 1). Racemic mixtures were used in the case of molecules that had chiral carbons. These odours are present in flowers and some in pheromones (see Table 1). Pure odorants (4 μl) were applied to 1-cm^2^ filter paper pieces, which were transferred to 1-ml syringes, cut to 0.7 ml to make them fit into the odour cannon. Fifty percent sugar solution was used throughout as US.

#### Experimental design

Our work was designed to obtain a generalisation matrix with 16 different odours. Ideally, after conditioning each of the 16 odours as CS, the response to each odour (including the CS) should be measured (i.e., 16 × 16 = 256 cells). However, testing 16 odours implies presenting them without reward, a situation that may result in extinction of the learned response due to the repeated unrewarded odour presentations. Preliminary experiments were performed in which four groups of 180 bees were trained along three trials to 1-hexanol, 2-octanol, linalool, and limonene, respectively. Training was followed by tests with the four different odours, including the conditioned one. These experiments showed that after three conditioning trials, the response of the bees to the CS in the four tests remained at the same level, independently of the order of occurrence of the CS such that it was not influenced by extinction. We thus kept this protocol for the 16 × 16 matrix. Each of the 2,048 bees used in this study was thus subjected to three conditioning trials with their respective CS, and to four test trials, each with a different odour chosen among the 16 possible odours. Intertrial intervals of 10 min were used throughout. A randomisation schedule (detailed below) was developed for the test phase to reduce any possible day- and odour-combination effects.

#### Conditioning trials

One bee at a time was placed into the conditioning setup. The total duration of each trial was 37.5 s After 15 s of familiarisation to the experimental context, the CS was presented to the bee for 4 s. Three sec after onset of the CS, the antennae were stimulated with the US, leading to a proboscis extension. The bee was allowed to feed for 3 s. Stimulus overlap was 1 s (interstimulus interval, 3 s). The bee was left in the conditioning place for 17.5 s and then removed.

#### Test trials

The procedure was similar to that for conditioning trials but no US was given after odour delivery. After the four test trials, PER to the US was checked once again. Animals unable to show PER at this point were not considered for the analyses. Overall, less than 2% of the bees died during the experiment, and less than 1% of the survivors showed no US reaction at the end of the tests.

#### Randomisation schedule

On each day, two to three experimenters worked in parallel, each training 16 bees at a time. In the training phase, the 16 bees were divided into four groups of four bees, and each group was trained to one of the 16 different odours. In the test phase, four out of 16 odours were presented to each of the 16 bees. The combination of four odours tested together changed in each experiment, so that any effect of having particular odours in the same test combination was suppressed. The whole experiment was planned in such a way that in any of our experimental groups, two given odours appeared at least once, but a maximum of three times together in a test sequence. This was possible by carefully picking out eight of the 16! (2.1 × 10^13^) possible experimental plans. Additionally, within each group, the testing order for the four test odours was determined randomly.

#### Data analysis and statistics

During the experiments, we recorded the response to the presented odour, that is, whether bees extended their proboscis after the onset of the odour and before the presentation of the sucrose solution in the case of reinforced trials, such that the anticipatory response recorded was due to the odour and not to the US. Multiple responses during a CS were counted as a single PER. The percentages of PER recorded during acquisition were used to plot acquisition curves (see Figure 1). To test whether bees learnt the different odours in a similar way, ANOVAs for repeated measurements were used both for between-group and for within-group comparisons. Monte Carlo studies have shown that it is permissible to use ANOVA on dichotomous data only under controlled conditions [68], which are met by the experiments reported in this study: equal cell frequencies and at least 40 *df* of the error term. The α level was set to 0.05 (two-tailed).

To ensure that we analysed a true generalisation response in the tests, and hence built a true generalisation matrix, we kept only those bees which had actually learnt the CS (71% of the bees used in this work). We therefore performed new analyses that only included those bees that responded to the CS before the presentation of the US in the third conditioning trial. A lack of response to an odour in the tests could be due either to the fact that the bees had not made any association between CS and US or because their motivational level was low. For all odours tested, we observed that responses to the CS in the third conditioning trial were equivalent to responses to the CS in the tests (McNemar test; see Results). We represented the responses of the selected bees to the test odours (see Figure 2). As the numbers of bees were now heterogeneous in the different groups, we could not use ANOVAs to analyse the responses in the tests (see above). We thus used χ^2^ tests for all further between-group comparisons. In the case of multiple two-by-two comparisons, the significance threshold was corrected using the Dunn–Sidak correction [α′ = 1 − (1 − α)^1/k^ where *k* is the number of two-by-two comparisons in which each dataset is used] in order to reduce the type I errors. Alpha values between α′ and 0.05 were considered as near significant.

#### Olfactory space

To observe the relationships between odours in a reduced number of dimensions, we performed a PCA, which identified orthogonal axes (factors) of maximum variance in the data, and thus projected the data into a lower-dimensionality space formed of a subset of the highest-variance components. We calculated the three factors, which accounted for most of the observed variance. Calculating distances between odours in the resulting putative olfactory space allowed the evaluation of their perceptual similarity, not only based on direct generalisation between these odours (i.e., generalisation from odour A to odour B and vice versa), but also including responses to these odours after conditioning to other odours (e.g., C, D, E, etc.). We performed cluster analyses to group odours, according to their respective distance in the olfactory space, using both Euclidian and city-block metrics, with Ward's classification method. Both metrics gave very similar results, so we later used only Euclidian metrics. Euclidian (i.e., direct) distances in the 16-dimensional space are defined as



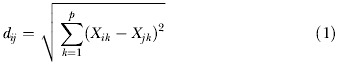



with *i* and *j* indicating odours, *p* the number of dimensions—that is, conditioning groups—and *X_ik_* the response of bees to odour *i* after conditioning to odour *k.* These distances were used in correlation analyses with optical imaging data (see below).

#### Correlation analysis between perceptual and optophysiological similarity measures

We studied whether or not physiological similarity between odours as determined by optical imaging studies of AL activity [22,23,35] actually reflects perceptual odour similarity for the bees. To this end, we performed correlation analyses between published optical imaging data that were obtained using the same set of odours as in our work [23] and our behavioural data. We used two sets of physiological data. First, to perform such a correlation on the whole dataset (including all 16 odours), we transcribed the activation maps presented by Sachse et al. [23] (see Figure 7) into activation levels for each glomerulus from zero to three, according to the following signal scale: dark blue (0%–20%) and light blue (>20%–40% activity), zero; green (>40%–60% activity), one; yellow (>60%–80% activity), two; and red (>80% activity), three. As the activity under 40% was less accurately separated from noise, activation levels between 0% and 40% were ranked as 0. Scaling the physiological data in this way instead of using the original imaging activation data, gave a good overview of physiological similarity between odours for imaging data (see Results). To provide a more precise correlation analysis between behavioural and imaging data, albeit on a more limited odour dataset (eight odours), we used exact correlation data ([23], Table 1). Each correlation value C, as calculated by Sachse et al. [23] between activity patterns for all pairs of primary and secondary alcohols, was converted into physiological distances by the operation 100 − C. All linear correlations were assessed by calculating Pearson's *r,* and using Student's *t*-test. Comparison between correlation coefficients obtained with the two methods was carried out statistically using a *Z* test as in [69].

## Abbreviations

AL: antennal lobe
ANOVA: analysis of variance
CS: conditioned stimulus
PCA: principal component analysis
PER: proboscis extension reflex
PNs: projection neurons
US: unconditioned stimulus
